# Fabrics and Garments as Sensors: A Research Update

**DOI:** 10.3390/s19163570

**Published:** 2019-08-15

**Authors:** Sophie Wilson, Raechel Laing

**Affiliations:** Materials Science and Technology, University of Otago, Dunedin 9016, New Zealand

**Keywords:** textiles, performance, electrical conductivity, end-use requirements

## Abstract

Properties critical to the structure of apparel and apparel fabrics (thermal and moisture transfer, elasticity, and flexural rigidity), those related to performance (durability to abrasion, cleaning, and storage), and environmental effects have not been consistently addressed in the research on fabric sensors designed to interact with the human body. These fabric properties need to be acceptable for functionalized fabrics to be effectively used in apparel. Measures of performance such as electrical conductivity, impedance, and/or capacitance have been quantified. That the apparel/human body system involves continuous transient conditions needs to be taken into account when considering performance. This review highlights gaps concerning fabric-related aspects for functionalized apparel and includes information on increasing the inclusion of such aspects. A multidisciplinary approach including experts in chemistry, electronics, textiles, and standard test methods, and the intended end use is key to widespread development and adoption.

## 1. The Review in Context—Purpose, Scope, A Focus on Fabrics

The purpose of this review is to address the gap that exists in understanding fibers, yarns, fabrics, and apparel that form part of wearable technologies, specifically fabrics and garments as sensors. Interactions between the functionalized apparel and the human body in which transient conditions are experienced are highlighted. This is achieved by critically reviewing the published scientific literature on electrically conductive fabrics/fabric sensors. Given that wearable technology is one of the fastest-growing areas in clothing and textiles, a review focused on this fabric/garment-specific area is timely.

While several reviews related to wearable technologies have been published since the beginning of the 21st century, their foci differed from the aim of the present review (e.g., a broad summary and overview [1,2,3,4,5,6,7,8,9,10,11,12,13,14,15,16,17,18]; applications and/or end-use—for example, tennis [19], medical care [20], electrocardiographic monitoring [21], neurobiological rehabilitation [22], gait recognition [23], a smart textile suit [24], flexible lithium batteries [25], motion sensors [26], and human activity monitoring [27]; properties of functionalizing substances, including electrically-conductive polymers [28,29,30] and carbon-based materials [31,32,33,34]; processes for functionalizing, such as e-broidery [35]; and methods to determine performance properties [36,37,38]). Each of these reviews is written from a perspective other than fabrics. Where fabric has been considered, the differences in structure and fiber composition have been highlighted [5]; however, the effects on the performance properties of fabrics and apparel are often not determined.

This review is organized into sections on fabric structure, processes for applying functionalizing substances to yield electrical conductivity, and properties essential for apparel. Being based on peer-reviewed published scientific papers and book chapters, the review represents up-to-date knowledge. The published literature reveals a need to focus on fabrics and methods to determine the performance of functionalized fabrics. The review excludes self-contained devices (e.g., sensors, actuators, and batteries) integrated in apparel/textiles; functionalized fibers and yarns not processed to fabrics; and issues related to data production, collection, transmission, and management. Fabrics, electrically-conductive substances, application processes, and designated end use identified in the research are described in Table 1 and referenced throughout the review. (For convenience, key terms related to textiles for wearable technologies are defined in Table S1).

## 2. Fabrics—The Effect of Fiber Composition and Fabric Structure

Fabrics composed of natural or synthetic fibers are typically electrically insulating, and therefore require nontextile additions to become electrically conductive [161]. Fiber composition has been reported to have an effect on the electrical properties of fabrics despite differences in structural properties, including yarn diameter and fabric density. Woven fabrics of cotton, viscose, silk, wool, and polyester had an electrical resistivity of 1.76 × 10^12^ Ω/m, 3.62 × 10^11^ Ω/m, 2.40 × 10^13^ Ω/m, 2.30 × 10^13^ Ω/m, and 4.35 × 10^13^ Ω/m, respectively, at 35% relative humidity (RH) [114]. Structural properties also affect electrical properties, thus for such comparisons of fiber composition, all else should be the same except the property of interest. Details of fabrics that have been functionalized are given in column two of Table 1. Where relevant, units have been converted to SI, with those used in papers reported in brackets. Some descriptions of fabrics were incomplete. Omitting this information limits our understanding of fabrics critical to the resulting performance, and comparability among findings becomes difficult. 

For fabric sensors, synthetic fibers (e.g., polyester, polyamide) and cotton fibers are most frequently used, with other natural fibers (e.g., silk, wool) and manmade fibers (e.g., viscose) used less (Table 1). Some investigators suggest the prominence of fiber type for apparel as the reason for selection (e.g., cotton, polyester [84,170]). Both wool and cotton can be considered high-end/expensive fibers [171], which may contribute to their limited use. The costs of production and end-user purchase become issues when the market sector is low-cost wearable technologies in mass production. Cost is not a defining factor for high-value sectors including medical and work safety. The inclusion of some form of elastane in fabrics/garments is desirable to increase the dimensional stability and therefore enhance sensor functionality by minimizing interference/noise. Moreover, if an electrically-conductive component moves as the fabric does, this may reduce displacement and degradation [172]. 

Electrical conductivity can be imparted to fibers, yarns, fabrics, or garments/apparel. Functionalized fibers and yarns need to withstand processing operations to fabrics and then garments that may damage (i.e., crack, break) them or remove the coating. For example, spinning and knitting have been shown to reduce treatment coverage [127]. Functionalizing fibers and yarns could result in superior fixation by penetrating the molecular structure, something difficult to achieve when functionalizing fabrics. However, subsequent processing and therefore potential damage is more or less eliminated when functionalizing fabrics. Most wearable applications require the functionalized textile to be in the form of a fabric or garment rather than a fiber or yarn to be worn on the body. 

Woven structures are more common than knits, with nonwoven used much less (Table 1), perhaps due to the greater physical stability of interlaced yarns of wovens compared to interlocking loops in knits. Structural stability facilitates functionalizing fabrics and influences the use of these fabrics. However, knits are desirable for many wearable applications because of flexibility and elasticity. Plain and twill weaves are the most common examples of wovens, and satin structure is also sometimes used. In terms of knit fabrics, a common descriptor is weft knit, and single jersey and interlock are frequently used. Lesser use of nonwovens could be related to the relatively poor flexibility and elasticity, which accounts for their minimal use in apparel [173], but nonwovens do have potential for other applications in sensing (e.g., geotextiles [174], membranes [175], filters [176]).

Fabric properties relevant to an end application require consideration. Where available, these are listed in column two of Table 1. Fabric structure and fiber type are most commonly included, with fabric mass, thickness, and stitch density sometimes also specified. Descriptions with missing information, especially structure type and clarity in function, or those that are unclear are identified by the relevant symbol. The mass of woven fabrics ranged from ~60 g/m^2^ to 350 g/m^2^, with the most common values between 100 g/m^2^ and 200 g/m^2^; thickness values were between 0.1 mm and 0.4 mm, most commonly close to 0.4 mm, and the number of warp yarns (11 to 55) was typically greater than the number of weft yarns (10 to 60). In terms of knit fabrics, the mass was ~100 g/m^2^ to 380 g/m^2^, the thickness was ~0.4 mm to 0.7 mm, and of the five studies that included stitch density, the number of wales exceeded the courses in three. Therefore, the knits typically were heavier and thicker than the wovens. Other properties included linear density, yarn fineness, and fiber length. For nonwoven fabrics, only the fiber content was described, and there were some instances where the fabric structure was not given, i.e., only the fiber type was provided.

Electrically-conductive components can be applied in discrete locations or positioned in a sensor array: across the width, down the length, or in a grid structure over a garment [69]. To achieve specific applications (e.g., monitoring dynamic properties such as motion and physiological changes over time), the position of the sensor relative to the human body site is critical. Diversity in human size, shape, and proportion complicates both the sizing and the positioning of these sensors, with the risk of displacement [177,178]. The presence of elastane can improve the stability of sensor placement on the body [177]. Considering the target population, anthropometric requirements have been highlighted [177] and are essential for the successful development of wearable technologies. 

## 3. Electrically Conductive Materials—Types and Incorporation Processes

### 3.1. Metal Filaments

#### 3.1.1. Yarn Structure

Metal filaments including stainless steel and copper can be incorporated in yarns and fabrics to produce sensors as each has high inherent electrical conductivity, although these metals may exhibit properties incompatible with textiles (i.e., flexural rigidity, elasticity) [10,126]. Three structures of metal yarns for incorporation into fabric were identified:

1. Metal filaments only, spun in monofilament and multifilament yarns (e.g., Bekintex, a 100% spun continuous cold-drawn stainless-steel yarn, 1 Ω/cm [35]);
2. Metal filaments twisted with textile fibers/filaments in ply yarns. This process can decrease flexural rigidity and increase elasticity, albeit with lower electrical conductivity than 100% metal yarns (e.g., 5 KΩ to 10 KΩ for 150 cm length of 20% stainless/80% polyester [49]; and 40% polyester/40% copper/20% stainless steel [118] were electrically conductive);
3. Core and covered assemblies with metal filaments for the core, (e.g., stainless steel wrapped in silk fibers [43], copper wrapped with cotton in blends of 63% copper/33% cotton, 80% copper/20% cotton, and 90% copper/10% cotton [48]); and metal filaments used to cover textile cores (e.g., monofilament silver-plated copper twine with polyester core [49], nylon core wrapped with three stainless-steel filaments and metal clad (silver, nickel, copper, gold, tin) with aramid DuPont^®^ core [35], and two stainless-steel yarns twisted around viscose yarns [120]).

#### 3.1.2. Fabric Construction 

Metal filaments can be incorporated in warp/weft or wales/courses in wovens and knits, respectively (Table 1). Shieldtex^®^ yarn (silver-coated polyamide) seemed common for integrating in fabrics and for embroidery. Tubular intarsia knitting was frequently identified, desirable due to the seamless knitting minimizing discontinuity caused by stitched seams, which can disrupt electrical transmission. Intarsia fabrics were often double-faced to protect electrically-conductive yarns between the two faces from exposure to light, moisture, and abrasion. However, the increased thickness of the double layer may be unacceptable for some wearable applications, particularly when worn next to the skin.

Yarns are exposed to tension and abrasion during fabric construction, and metal filaments may not be resistant to these. However, yarns of copper (40 μm) twisted with polyester were processed in a plain weave [2] and twist drawn 316 L stainless-steel filaments and woolen yarns were processed with a flatbed knitting machine and hand knitting [40] to yield electrically-conductive fabrics (Table 1). Unfortunately, some experimental details and evidence to support claims were missing. One example provided evidence of fabric (knit, structure not described) exhibiting lower electrical resistivity compared to the yarns (20% stainless steel/80% polyester yarn) from which they were manufactured [49] (Table 1). The reduced electrical resistivity of fabric was attributed to more contact points between staple fibers in the fabric than in the yarn [49]. The use of electrical resistivity makes comparisons between yarns and fabrics acceptable because area is considered. However, the narrow linear dimensions of yarns compared to complex planar structures of fabrics should be considered.

Differences in electrical conductivity can be related to the direction of integration. Differences in performance between wale and course are evident: one investigation suggested that the course direction has greater stability [118], while another suggested greater stability in the wale direction [120] (Table 1). Knit structures do vary in terms of the stability, the structure itself (i.e., single jersey, interlock, rib), and whether a warp or weft knit will affect the fabric properties.

#### 3.1.3. Embroidery

Yarns of 100% metal composition can be difficult to embroider. Yarn flexural rigidity causes challenges for manipulation through mechanics of machine sewing, threading needles, and sewing through fabric [35]. For instance, a 100% stainless-steel yarn (Bekintex) cannot be sewn using embroidery techniques, but a couching technique can be used to fasten to fabric [35]. A variation of this yarn (Bekintex 15/2) can be machine embroidered with constraints of the short length of staple fibers causing short circuits [35]. Other stainless-steel yarns with a very fine diameter (e.g., 100 µm, 12 µm, 2 µm) can be machine sewn [35]. Specialized machines or settings may also help minimize potential damage to electrically-conductive yarns and fabrics.

Fabric sensors have been successfully developed with embroidery techniques by blending metal filaments with textile fibers (Table 1). A nylon core wrapped with three stainless-steel filaments can be sewn with an embroidery machine [35]. Silver-plated nylon yarn (140/17 dtex) was embroidered on cotton fabric (0.43 mm) and demonstrated to perform as a humidity sensor between 25% and 65% at 20 °C based on changes in impedance [56]. Subsequently, the same researchers determined that Shieldtex^®^ 117/17 dtex 2-ply (polyamide and silver) had lower sensitivity to humidity (30% to 65%) than Bekaert yarns of 80% polyester/20% stainless steel, 80% cotton/20% stainless steel embroidered on 100% cotton (0.43 mm) [54]. 

Circuits to facilitate sensing have also been made of embroidered yarns. For example, conductive yarn was embroidered on 100% cotton satin, followed by drop coating of polymer and single-walled carbon nanotube solutions, and showed responsiveness in electrical resistance to volatile compounds (e.g., ethanol, pyridine, methanol) [58]. No reference to the effects on flexural rigidity nor to the elasticity of the resulting fabric were included, but embroidery is a possible integration process.

Factors that interfere with sensor functionality, such as manufacturing variability, have been explored. Shieldtex^®^ 117/17 dtex 2-ply embroidered on cotton fabric (0.43 mm) with a satin fill stitch pattern (Singer Futura XL-550 embroidery machine) was reported with an error of up to 6% in impedance measured from 25%RH to 65%RH over 10 specimens [55]. If the textile sensors are designed for use in critical applications such as health care and safety, scrutiny of the variability in sensor performance is essential. Such investigations into manufacturing variability would also be useful for other functionalisation processes (e.g., fabric treatments).

### 3.2. Fabric Treatments

#### 3.2.1. Intrinsically-Electrically-Conductive Polymers

Intrinsically-electrically-conductive polymers offer another option for conferring electrical conductivity (<10^−10^ S/cm insulators, <10^−5^ S/cm semi-conductors, 10^2^ S/cm conductors), attributable to the conjugated chemical structure and additives (dopant, monomer, oxidant) [5,10,30]. Intrinsically-electrically-conductive polymers exhibit challenging properties including brittleness, comparatively poor flexural rigidity, and poor resistance to abrasive processes [10,30]. Combining with textiles can ameliorate some poor performance properties [30], although durability is an ongoing challenge.

In terms of fabric application, many polymerization processes have been used, and intrinsically-electrically-conductive polymers were more commonly applied to wovens than knits to confer electrical conductivity (Table 1). The efficacy of polymer application is related to the surface to which it is applied, emphasizing the importance of both the fabric structure and the fiber composition. For example, of 14 woven fabrics treated by vapor deposition of poly(3,4-ethylenedioxythiophene), those with high porosity had lower electrical resistance and the fiber composition of the fabrics could be ordered from lowest to highest: bast, cotton, silk, linen, wool, and rayon [75]. However, separating the effects of fiber type and porosity is not possible with unmatched fabrics, i.e., multiple fabric parameters differed. The same trend of fiber type was demonstrated with 760-mm (3”) yarns extracted and treated by vapor deposition of poly(3,4-ethylenedioxythiophene) [75], although the yarn properties could also differ (i.e., twist, ply). Testing fabrics of the same fiber composition and base structure but with different porosity would provide a useful comparison.

#### 3.2.2. Carbon-Based Substances

Carbon-based substances are desirable to impart electrical properties to fabrics, since they are a natural source and exhibit high electrical conductivity (e.g., 10^4^ S/cm) [7]. Carbon is most commonly applied in the form of carbon nanotubes, single- or multi-walled. Different processes to functionalize fabrics with carbon include dipping [84,86] and screen printing [84]. Carbon has been applied to woven fabrics [79,80,81,82,84,86] and, much less frequently, to knit fabrics [125,133] (Table 1). However, one fabric is referred to as knit, whereas the image appears to be woven [133], which illustrates scant attention to fabrics. Carbon-loaded rubber has also been reported to have acceptable performance for sensing garment prototypes, similar to that of existing sensor devices (e.g., WEALTHY [120], a piezoresistive sensor [42], and the Arctic project [116]). No evidence of carbon-treated fibers or yarns subsequently processed to fabrics was found.

Graphene is desirable to yield electrically-conductive fabrics, notwithstanding the high cost and small production volumes [179]. However, reduced cost is evident since first use, and growing demand for graphene may lead to increased production and decreased cost [180]. One example of the integration of cotton yarns treated with graphene constructed in a knit (interlock of nylon filament double-covered Lycra^®^) was identified, imparting electrical conductivity and responsiveness to changes in temperature [135]. A comparison between fiber and fabric treated with reduced graphene oxide was also identified [155]. Reduced graphene oxide was applied to wool and glass fibers, and aramid, polyester, cotton, and nylon fabric [155]. The measurements were reported as per cm, which improves the comparability, but structural differences between yarns and fabrics are apparent, i.e., narrow linear substrate of fibers in yarn form and a complex planar structure of fibers and yarns in fabric form.

In terms of fabrics, graphene has been applied to wovens and knits composed of cotton, polyester, polyamide, wool and also to some nonwovens (Table 1). Graphene application to fabric involves three main steps: i) oxidization of graphite to produce graphene oxide (Hummers method); ii) application of graphene oxide to fabric; and iii) reduction reaction (chemical, thermal, ultraviolet light). Published investigations indicate that application followed by reduction is an effective process. However, a publication in 2017 suggested that reducing graphene oxide in solution with sodium hydrosulfite followed by subsequent pad-dry application to 100% cotton 3/1 twill was more efficient to produce electrically-conductive fabrics while maintaining a cost-effective process [94] (Table 1).

## 4. Properties of Fabrics Functionalized for Electrical Conductivity

### 4.1. Key Properties

The efficacy of sensor performance is determined by the change in electrical conductivity after exposure to an agent (e.g., volatiles [58], human body parameters [42,43]); the effect on impedance and capacitance can also be measured. In vitro investigations are common, with in vivo studies carried out much less frequently. Undertaking sequential in vitro and in vivo investigations is useful to demonstrate performance. Experimental work is mostly proof of concept evidenced by laboratory tests, including electrical conductivity [115], impedance [137], and/or capacitance [110]. Typically, the incorporation procedure is explained, as are lab-based phases of some performance properties. The evidence of treatment deposition is frequently quantified, specifically microlevel presence, including atomic force microscopy [110], scanning electron microscopy [93,97,115], transmission electron microscopy [166,167], Raman spectroscopy [106,142,181], Fourier transform infrared spectroscopy [93,106,142,181], and X-ray diffraction spectroscopy [106,181]. Proof of concept studies are important; however, expanding knowledge of performance over time and related to end use is required if the products are to reach commercial applications.

The conventional properties of apparel and apparel fabrics need to be retained, a point that is not often taken into account. The issue is whether the change in function is practically important (e.g., whether changes in properties are detectable instrumentally or perceptible when used by humans). Key properties for apparel include thermal and moisture transfer, elasticity, flexural rigidity, resistance to abrasion, care treatments, and environmental effects in use and disposal. Drape is related to the form of the fabric when worn on the body, related to flexural rigidity and elasticity, and not considered in research on fabric sensors. Apparel fabrics are not routinely exposed to high levels of tensile stress compared to fabrics which may be used for applications such as parachutes or harnesses. Therefore, tensile breaking strength is not considered a key property. However, repeated small extension and relaxation cycles, i.e., elasticity, are important.

Standard test methods are preferred unless there is a good reason for not doing so. Table S2 lists the standard test methods for measuring properties relevant to apparel and apparel fabrics, and gives examples of their use in published papers. International standards are more desirable than national or regional standards because of the wide acceptance. 

### 4.2. Measurement of Electrical Resistance

Table 2 gives the standard methods available to determine the electrical properties of fabrics, developed by the European Organization Supporting Standardization for Smart Textiles (SUSTASMART) [38], the American Society for Testing and Materials (ASTM) [182], and the European Committee for Standardization [183]. Throughout the review, findings related to electrical conductivity are reported as they appear in the published work, i.e., measurement units are not converted. 

Evidence of use of standards in published research was sparse; instead only the instrument and technique is described (e.g., multimeter). The two-probe method is often used to measure yarns or narrow strips of fabric and the four-probe method for larger specimens. A recognized challenge of the two-probe method is that the measurement includes the electrical resistance of the textile, leads, and connectors, which typically varies among instruments [10]. A four-point measurement can ameliorate this [184]. Consensus in terminology (i.e., electrical conductivity or resistance) and units would be useful. Electrical conductivity is usually referenced, but supported with information about electrical resistance; sometimes it is converted to the electrical resistivity considering area (square, length, width, mass, and/or thickness). 

In laboratory experiments, relative humidity and temperature are not always reported and/or controlled. This is important due to the effects of temperature and moisture presence on electrical properties as well as on many fiber types. For example, a change in humidity (25% and 65% at 20 °C) caused a change in the impedance of cotton fabric (0.43 mm) with embroidered silver-plated nylon yarn [56]; and a graphene/methyl-red composite and silver nanoparticles inkjet printed on polyethylene terephthalate showed reduced electrical resistance with increased relative humidity [191]. In terms of changes in temperature, the change in electrical conductivity of a graphene-based sensor in response to carbon dioxide differed when conditions of 40 °C and 60 °C were compared to 22 °C [192], and a graphene nanowall film on polydimethylsiloxane increased in electrical resistance from 706.2 Ω at 25 °C to 98.04 KΩ at 120 °C [193]. The effect differs depending on the moisture absorption and heat conduction properties of the textile and electronic materials. Experimental work on textile fibers, yarns, fabrics, and apparel needs to be carried out under controlled conditions, or the conditions at least need to be monitored and reported. Additionally, wear trials are limited and the transient conditions seem not to have been considered. 

### 4.3. Apparel-Specific Properties

#### 4.3.1. Thermal and Moisture Transfer

The environment, metabolic activity, and work of humans contribute to heat production. Apparel resists transfer of heat and moisture compared to a noncovered body, thus affecting homeostasis [194]. Textile variables with reported effects on thermal and moisture transfer include fiber diameter, type, hydrophobicity/hydrophilicity, and length [195]; yarn twist and diameter; fabric construction, i.e., size and number of interstitial spaces, thickness, and surface treatments [7,194,195,196]; garment fit, air spaces, and layering [197]. All textile fibers have similar thermal conductivity, with the presence of air having the greatest effect [196]. The effect of apparel and fabric structure overrides that of fiber type [197]; fiber type has an effect when moisture is present [197].

Six studies demonstrated the effects of electrically-conductive coatings on thermal and moisture transfer (Table 3). The composition of treatments, fabric structure, fiber composition, and methods to determine performance varied, and the trends in performance were inconsistent. Permeability to air, permeability to water vapor, and water retention decreased after functionalization in two investigations [93,131], while another study reported minimal changes in permeability to water vapor and air following functionalization [115]. Thermal conductivity was reported to increase in a different study [65]. Changes can be attributed to electrically-conductive treatments encapsulating the fabric, yarn, and/or fiber, causing changes to interstitial spaces [93,115], and fabric thickness [131].

Of the standard test methods, some may be more suitable than others. The applicability of ASTM D6767-14:2014 and ISO 8096:2005 to the thermal transfer of fabrics is not known given that the intended application of the fabric was not given [93]. Because properties measured were known to be relevant to human physiology (e.g. thermal and moisture transfer), wearable applications were assumed, thus using a method related to geotextiles may not be applicable. Also, the fabrics were not rubber- or plastic-coated as specified in the method, but treated with graphene oxide. In the absence of more specific methods, the difference of treatment type could be acceptable. ISO 11092:2014 Textiles‒Physiological effects‒Measurement of thermal and water-vapor resistance under steady-state conditions (sweating guarded hotplate test) may be more acceptable [198]. Permeability to water vapor was measured with a cup method, and water retention determined with an immersion technique [131] rather than a standard test method such as ISO 11092:2014 Textiles‒Physiological effects‒Measurement of thermal and water-vapor resistance under steady-state conditions (sweating guarded-hotplate test) and BS 7209:1990 Specification for water vapor permeable apparel fabrics (Table S2.) [198,199]. However, detailed descriptions of the methods used were provided, which is especially important for understanding and potentially replicating such an investigation.

#### 4.3.2. Elasticity and Flexural Rigidity

Elasticity (extension and recovery) and flexural rigidity are critical properties for fabrics worn close to the body with continuous multidirectional changes [200,201]. No fabrics are totally elastic (i.e., viscoelastic, creep [202]). Several textile variables affect elasticity: fiber type, i.e., chemical and molecular structure, and inherent crimp [203,204]; yarn twist and presence of elastane [42,118,125,205]; fabric structure [200,206]; and garment construction, principally seams and layering [200].

Electrically-conductive treatments can decrease the elasticity of fabrics due to adhesion between fibers and yarns caused by increased diameter and/or filling interstitial spaces. Some reduction in elasticity may be acceptable for outerwear, but less so for next-to-skin apparel. The effects of electrically-conductive treatments on elastic performance of fabrics seem not to have been considered. Investigations on extension and sometimes recovery relate to the performance and reliability of stretch sensors [41,125]. Extension and recovery can cause cracking or distortion of treatments, negatively affecting electrical conductivity. However, surface cracking has been used as a measure of change in electrical resistance for strain sensing functions due to separation and connection with extension and recovery, respectively [141,142].

Functionalizing fabrics with electrically-conductive treatments can lead to a reduction in fabric bending. Flexural rigidity, shear modulus, and force hysteresis, measured with the fabric assurance by simple testing (FAST) method and the Kawabata evaluation system for fabrics (KES-FB), increased following polyurethane treatment on three-layer weft knits constructed of stainless steel/polyester and carbon core/polyester yarns [131]. FAST and KES-FB are established, but not standard methods nor widely adopted. Thus, investigations of the effects on fabric flexural rigidity are limited in number and scope.

The effect of repeated bending on electrical properties has been investigated. For instance, stainless-steel yarns treated with reduced graphene oxide, manganese dioxide, and polypyrrole maintained capacitance (80%, 91%, 103%) following deformation tests (1000 90° bends, knots, twists, respectively) [40]. Degradation of ~20% capacitance and reduced electrical conductivity of carbon-coated plain weft knit and plain woven carbon fiber patches occurred following repeated bending at 90°, 135°, and 180° while fastened to a hinged wooden plank [172]. The authors suggested this was caused by loosening and/or delamination of a carbon coating [172]. An angle of 90° was used, but the other bending techniques and angles differed and therefore the results are not directly comparable. Repeated cycles related to the end use are desirable to understand the performance during use. Investigations with standard methods would enhance the determination of performance.

A change in electrical conductivity with extension/recovery and bending is desirable for detecting changes if these are the properties of interest (e.g., for body movement, respiration, heart rate). However, when sensing other parameters (e.g., the presence of gas, a change in humidity and/or temperature), the changes in electrical conductivity can confound the response, providing misinformation. This is a challenge that seems not to have been addressed. Changes can also indicate degradation if the electrical properties do not recover. 

### 4.4. Effects of Use

#### 4.4.1. Resistance to Abrasion

Abrasion can damage fabrics by fibrillation, yarn breakage, holing, unacceptable appearance (e.g., fuzzing and pilling), and an overall reduction in strength. Damage to fabrics functionalized with electrically-conductive substances can be due to treatment displacement and removal or inherent degradation, i.e., cracking. As a result, the electrical conductivity is typically reduced.

Poor resistance to abrasion was evident in most investigations. Graphene-treated knit and woven fabrics (140 g/m^2^) increased from 6.47 MΩ/square and 7.92 MΩ/square to 10.96 MΩ/square and 13.30 MΩ/square, respectively, after dry rubbing; following wet rubbing they increased by 0.61 times and 0.67 times, respectively [93]. Poly(3,4-ethylenedioxythiophene)-treated 100% polyester woven fabric increased from 1.0 KΩ/square to 1.5 KΩ/square or 0.6 KΩ/square to 1.9 KΩ/square with and without fluorinated decyl polyhedral oligomeric silsesquioxane following 10,000 abrasion cycles as per ASTM D4966 [76]. Poor resistance to dry and wet rubbing has also been reported for polypyrrole-treated knit of 20% treated/80% nontreated, and 100% treated fibers (structural information was omitted) [126]. These properties were determined in accordance with ISO 105X12: 2016 [207]. The use of a standard test method is desirable; however, ISO 105X12: 2016 is a test for color fastness and thus not directly applicable to electrically-conductive treatments. The Martindale test, in accordance with ISO 12945-2:2000 for pilling and ISO 12947-2:2016 for abrasion, is possibly more acceptable. Abrasion with the Martindale test resulted in the increased electrical resistance of 100% wool woven treated with poly-3-decanylpyrrole [61]. Including information related to the number and type of cycles would be useful to indicate performance.

Non-standard test methods have also been used in investigations. For example, polypyrrole-coated woven craft store fabrics differing in fiber content (wool, cotton, linen, silk, bamboo rayon, pineapple and banana fiber bast) have been shown to retain electrical conductivity following rubbing with bare hands and scraping with the sharp end of a metal spatula [75]. This is an example of low-intensity rubbing compared to the standard methods, and is less desirable due to the limited applicability to end-use practices, control, and repeatability.

#### 4.4.2. Cleaning Treatments

Cleaning of fabric sensors is complex due to the material composition, including fibrous assemblies (i.e., fabric structure, layers), electronics, polymers, and other treatments. Failure of one component could result in failure of the assembly, consequently cleaning needs to be appropriate for the apparel as whole, not each separately. In cleaning, there are several options including water volume, wash temperature, duration, spin speed, and detergent. Selection of these is governed by fiber and fabric type and, to some extent, garment type. Multiple washes for repeated use and performance over time are useful because the effect of the first wash often differs from that of subsequent washes. Repeated wash cycles were not common.

Examples of investigations to determine the durability to cleaning (washing or dry cleaning) are described in Table 4. Poor fastness was common, evidenced by increased electrical resistance. Some researchers claimed durability to cleaning, although the evidence provided was typically inadequate. The results included in Table 4 are reported as they are presented in the papers. The methods used are varied (some standard methods and some others), and comparability among studies is thus limited. Sparse information was provided, so including the details of the cleaning cycles of both standard and other methods is useful. 

Methods for cleaning often include dipping in water or a detergent mixture, mechanical agitation, rinsing, and drying [47,50,75,86,92,97,98,120,164] (Table 4). Some apparel requires hand washing, but machine washing is more common. In comparison to standard methods for machine washing (e.g., ISO 6330, ISO BS EN 105 C06, ISO 105-C03, AATCC 61-2A, AATCC 132, AATCC 86), these techniques were low-intensity and less representative of normal practice for apparel. Durability to machine washing has been tested following standard methods, international [45,77,93,94,115,116,122,126,127,146] and national [73,76,90,109], as well as nonstandard methods [47,91,162]. Using a device such as a thermal magnetic stirrer [96,138] has merit in terms of the controlled mechanical agitation (i.e., revolutions per minute), the size of the spinning rod, and the temperature. Small specimens are typically investigated, and controlling the agitation is difficult; the use of small-scale simulation permits control. Where possible, the process should be mechanized.

#### 4.4.3. Storage

The effect and conditions of storage require consideration. Changes in electrical properties over time are difficult to estimate and depend on the conditions of storage, including temperature, relative humidity, and light [36,184]. Consistency in performance over time is essential for multiple-use and single-use products because of reuse, unknown shelf life, and logistics prior to use. Minimizing the change over time related to experiments is also important to avoid confounding effects, and thus needs to be considered when designing experiments. Hanging can result in dimensional changes due to the effects of mass of the fabric, typical of knit structures, so flat storage is desirable. Layering fabrics should also be avoided to prevent any compression effect.

Evidence of degradation of the electrical properties of functionalized fabrics over time has been identified, in which the effects of temperature, relative humidity, and light are not separated and the descriptions are incomplete. For example, the electrical resistance of polypyrrole-treated Lycra^®^ increased and the piezoresistive response decreased due to ageing (i.e., oxidation) [70]. Polypyrrole films doped with p-toluenesulfonate increased in stiffness and brittleness, and decreased in electrical conductivity, breaking strain, elasticity, and elongation following four months of ageing at room temperature [208]. Poly(3,4-ethylenedioxythiophene) treated pineapple fiber woven fabric has been reported to retain an electrical conductivity of 298 S/cm following six months’ storage (benchtop in air) [75]. Thus, measuring the properties immediately following application does not give a clear indication of the performance over time, and the extent of degradation over time will vary based on the materials. The effects of factors cannot always be discriminated due to omitting details and/or not controlling aspects of storage.

#### 4.4.4. Environmental Effects

Considering the potential dispersion of electrically-conductive substances in the environment during use (e.g., wear and cleaning) and at the end of life (e.g., reuse and disposal) is important. Negative effects on human health and ecosystems may be attributed to substances being released into the environment. Taking into account effects on the environment is timely given increased international interest in sustainability, i.e., managing the resources and waste associated with a growing population, and greater awareness of microfiber release from apparel and other textile products during use and when disposed of [209,210]. The added complexity of diverse chemical compositions of substances used to functionalize fabrics could heighten the risks associated with the pollution, absorption, dissemination, and consumption of contaminants [211].

Naturally-sourced fibers such as cotton and wool, are desirable in terms of biodegradability compared to petroleum-based fibers such as polyamide and polyester. Managing chemicals and outputs for the production of fibers, yarn, fabric, and apparel processing is a challenge [212,213]. Diversity of fiber composition is desirable because each has advantages and disadvantages for end-use applications and in terms of effects on the environment [214]. Various other factors such as longevity and frequency of use, care, and process of reuse/disposal require consideration. The investigation of methods that require fewer chemical processes to produce regenerated cellulose (cotton, flax, linen, hemp, bamboo) or discoveries of other sources (e.g., coffee, fish skin) may be of interest. Naturally-occurring microorganisms have been discovered to have the ability to break down and use manmade fibers as an energy source (e.g., *Aspergillus tubingensis* can degrade polyester polyurethane [215], *Ideonella sakaiensis* can use polyethylene terephthalate as a source of energy and carbon [216], and polyethylene (HDPE) can be degraded by *Achroia grisella* [217]).

Full lifecycle assessment and other investigations such as degradation with exposure to microorganisms over a period of time provide useful information, yet is a challenging task. Examples of lifecycle assessments focusing on electrically-conductive fabrics and smart textiles were identified [218,219]. Specifically, a lifecycle assessment (eco-costs/value ratio) of the manufacture, use, transport, disposal and effects of considering eco-design, especially at the early stages (e.g., conception, design), was undertaken with a functionalized next-to-skin fabric prototype, Vibe-ing, designed for vibration therapy [219]. Vibe-ing is rib knit apparel composed of merino wool (Greggio Millennium yarn), elastane, silver-coated yarns (Bekitex), and knitted Elektrisola lines with knitted pockets containing 3D printed cases to hold printed circuit boards and vibration motors [219]. The production phase was reported to have the highest impact, wherein the electronic components had a greater impact than the textile sections, but merino wool production also had a high impact [219]. Thus, selecting materials was considered a primary factor. The authors suggested changing to copper could reduce the eco-costs by 45% over the use of silver, and acryl could decrease the eco-costs by 22% compared to wool [219]. However, copper will affect the aesthetic properties, and acryl may increase the level of cleaning required and therefore increases the impacts of the in-use phase. Reducing materials was also considered, i.e., a 75% reduction of Elektrisola, for which functionality was still considered possible [219].

Challenges related to the eco-design of electronic textiles, specifically material efficiency, hazardous substances, product obsolescence, and end of life, have been explored in a review-style paper and workshops with people of industry related to electronic textiles [218]. The authors highlighted the potential of applying established eco-design principles (e.g., Design for Recycling), the importance of labeling to implement such processes, compatibility standards to reduce obsolescence, and the importance of considering environmental consequences at the concept/design phases of production [218]. Disposal of conventional textiles and electronics was discussed, as was the difficulty of handling the heterogeneous waste of the combined textiles and electronics [220].

Lifecycle assessment was also performed for sensing fabrics not intended for wearables, i.e., sensing floor [221], curtain fabrics [222]. A key finding was the disparity between the fabrics in use (e.g., functionalized fire retardant wool washed 25 times compared to silver-nanoparticle-treated polyester washed 100 times with different wash temperatures, changing the energy consumption and the potential release of substances during the wash) [222]. Moreover, when disposed of, the items cannot be considered municipal solid waste nor conventional textiles (e.g., incineration has benefits over being in landfill, but there are unknown effects of emission in the air from the additional functionalized substances) [222]. Therefore, a consideration of the specific materials used in fabric sensors is critical for determining the environmental effects of use and disposal, i.e., we cannot extrapolate about other fabrics based on functionalized materials, nor on conventional fabrics.

The introduction of certification requirements for quality control and reducing environmental effects is necessary. Effective disposal of fabric sensors of wearable technologies is a necessity to manage the increasing demand [37]. As of 2019, no certification requirements were identified for fabric sensors or wearable technologies made from textiles. Investigation and formation of rules and regulations regarding disposal could be performed by institutions such as Oeko-Tex and/or government organizations [37]. Legislation exists for the disposal of electrical equipment, including the Waste Electrical and Electronic Equipment directive established by the EU. However, the applicability of this legislation to textiles with electronic components is not clear. Challenges in conventional electronic routes have been suggested, including rejection at collection points, discarding by recycling companies, novelty of the electronic/textile assembly, and equipment issues (e.g., jam shredders, crushers, incompatible with separators) [220]. An alternative option is secondhand clothing, as the items are still wearable even if the functionalization is lost, especially if the electronic components are inconspicuous [220]. Functionalized apparel could also be handled by conventional textile routes; however, the electronic components may be incompatible with certain disposal processes (e.g., shredding) and thus could contaminate the output [220]. Manual separation is also an option, albeit a difficult one.

## 5. Conclusions

Omitting specifics related to fabrics, experimental details, and results of studies limits our understanding of the research findings. Despite this, some comprehensive descriptions and studies have been carried out that exemplify detail needed. Diverse processes for functionalization can be used with varying success to impart electrical conductivity. Minimizing the changes to apparel properties (i.e., changes to thermal and moisture transfer, elasticity, flexural rigidity), ensuring durability, and managing the environmental effects of production are ongoing challenges.

Standard test methods for determining fabric performance are rarely used and the acceptability of conventional fabric test methods is not clear given the addition of nontextile components. A multidisciplinary approach involving experts from the fields of chemistry, electronics, and textiles, and those involved in development of standard methods, is required to meet this challenge.

## Figures and Tables

**Table 1:** Investigations on producing functionalized fabrics. (^†^ not sufficiently defined, unspecified; * description unclear.).

Fabric Construction/Treatment Reference	Fabric Structure and Fiber Content				Functionalizing Material	Function
**Woven**						
**Constructed in Fabric**						
**Stainless Steel**						
[39]	•woven ^†^				•100% surgical stainless-steel mesh	•pH sensor
[40]	•woven ^†^				•316 L stainless-steel filaments twisted, treated with polypyrrole, manganese dioxide, reduced graphene oxide	•energy storage
[41]	•plain woven, polyamide/Lycra^®^				•Bekintex 50/2; Beag EA1088	•stretch sensor
[42]	•double face tubular intarsia				•stainless steel twisted around continuous viscose yarn	•physiological/biochemical sensor
[43]	•woven cotton ^†^				•stainless steel covered with silk	•ambulatory monitoring of physiological parameters
[44] (all values described in the article)	•4-end 2/2 twill 11.42 warp/10 mm to 13.78 warp/10 mm (29 ends/inch to 35 ends/inch); 11.81 weft/10 mm to 24.41 weft/10 mm (30 picks/inch to 62 picks/inch); mass ~114 g/m^2^ to ~520 g/m^2^; linear density 39.4 tex/1 to 163.2 tex/1				•open-end friction core-spun yarns stainless-steel filament, stainless-steel staple/polyester staple each used as cover and core, two ply yarn; stainless-steel filament core, stainless-steel staple/rayon staple cover; 100% stainless-steel staple yarn	•shielding home electronics, electrical appliances, cellular phones, digital devices
**Copper**						
[45]	•woven silk, hand loom ^†^				•copper polyamide substrate connected to touch sensor encapsulated with polydimethylsiloxane	•e-textile applications ^†^
[46]	•plain, twill, satin				•polybutylene terephthalate polymer wire coated with copper	•harvest solar and mechanical energy
[47]	•woven ^†^				•100% polyamide filament coated with copper in warp and weft, 52 ± 5 g/m^2^	•electromagnetic shield
[48]	•plain woven, 100% cotton				•DREF-3 friction spun 38 standard wire gauge copper filament core and MCU-5 cotton fiber cover in ratio of 67/33, 80/20, 90/10; warp and weft, one just weft	•electromagnetic shield, mobile phone charging, body temperature sensor
[49]	•woven ^†^				•Swiss Shield CUPES-L 54 nm (18.5 tex) core polyester fibers, monofilament silver-plated copper cover	•electrodes to monitor heart rate
**Silver**						
[39]	•woven				•argon mesh 100% nylon, 55% silver treated, ripstop 100% silver-coated nylon	•pH sensor
**Nickel**						
[50]	•woven, knitting wool *				•nickel/titanium filament covered with polyurethane	•sitting posture correction
**Conductive Yarn**						
[51]	•woven (textile yarns), conductive yarn warp, plastic fibers weft, inserted in cotton undershirt ^†^				•conductive yarn; plastic fibers with electrical components, i.e., circuits; temperature-sensitive chip, silicon based	•temperature sensor for healthcare
[10,52,53]	•woven in upper body garment ^†^				•optic fiber sensors	•monitor vital signs ^†^
**Embroidery**						
[54]	•100% cotton thickness 0.43 mm ^†^				Shieldtex^®^ 117/17 dtex 2-ply silver-plated polyamide Bekaert^®^ yarn 80% polyester/20% stainless steel, 80% cotton/20% stainless steel	•moisture sensor
[55,56]	•100% cotton thickness 0.43 mm ^†^				•Shieldtex^®^ silver-plated polyamide yarn (140/17 dtex)	•moisture sensor
[57]	•100% polyester ^†^				•reduced graphene oxide-coated nylon filaments, silver threads	•skin temperature sensor
[58]	•satin, 100% cotton				•conductive thread with drop coating of polymers (polyvinyl chloride, cumene terminated polystyrene-co-maleic anhydride, poly (styrene-co-maleic acid) partial isobutyl/methyl mixed ester, polyvinylpyrrolidone) and single-walled carbon nanotubes	•gas sensor
[59]	•plain woven, 100% cotton 20 warp/10 mm, 22 weft/10 mm (100 warp × 110 weft per 5 cm × 5 cm), mass 154.9 g/m^2^, thickness 0.39 mm; plain woven, 50% stainless steel/50% cotton 11.4 wales/10 mm, 9.8 courses/10 mm (57 wales × 49 courses per 5 cm/5 cm), mass 155.0 g/m^2^, thickness 0.44 mm				•100% stainless-steel yarn (100 f/2)	•motion sensor, electrodes
[43]	•woven, 100% cotton ^†^				•stainless steel covered with silk	•physiological parameters ^†^
[35]	•woven ^†^				•Bekinox^®^ BK 50/2 polyester/steel staple fibers; VN 140 nylon/35”3 (nylon core stainless-steel cover); Bekintex 100% stainless-steel filament; Bekintex 15/2 100% stainless-steel spun filament; metal clad cover (silver, nickel, copper, gold, tin), aramid core (Kevlar^®^)	•textile-based computing ^†^
**Chemical Treatment**						
**Polypyrrole**						
[60]	•plain woven, 100% cotton scoured, 52 warp/10 mm, 28 weft/10 mm (cm), mass 112 g/m^2^				•sequential chemical and electrochemical polymerization	•electrocardiogram sensor
[61]	•woven, 100% wool ^†^				•solution, vapor, spray polymerization, brush coating	•electrically conductive textile ^†^
[62]	•plain woven, 100% polyester				•vapor-phase polymerization in the presence of fluorinated alkyl silane	•multifunctional protective clothing and electronic textiles ^†^
[63]	•woven, Nylon 66 DuPont^®^ Type 200 mass 124 g/m^2 †^				•immersion	•electrically conductive textile ^†^
[64]	•2/1 twill, 100% wool mass 228 g/m^2^ plain woven, 100% polyester mass 212 g/m^2^				•solution polymerization	•electrically conductive textile ^†^
[65]	•pinstripe twill, 100% wool 32 warp/10 mm, 30 weft/10 mm (cm), mass 216 g/m^2^, thickness 0.48 mm; plain weave, 100% worsted wool, 30 warp/10 mm, 23 weft/10 mm (cm), mass 226.61 g/m^2^, thickness 0.89 mm				•chemical polymerization, physical vapor deposition	•electrically conductive textile ^†^
[66]	•woven, 100% polyester ^†^				•surface polymerization	•electrical stimulation to cells, biostability
[67]	•2/2 twill, 100% polyester 27.56 warp/10 mm (70 ends/inch), 21.65 weft/10 mm (55 picks/inch)				•polymerization in the presence of sulfosalicyclic acid	•electrically conductive textile ^†^
[68,69,70]	•100% Lycra^®^				•polymerization (with carbon filled rubber)	•posture, gesture, body kinematics
**Polyaniline**						
[71]	•plain woven, 100% polyester 29 weft/10 mm, 35 warp/10 mm (cm), mass 123 g/m^2^, nondyed, yarn linear density weft 167 dtex 48 filaments, warp 77 dtex 40 filaments				•chemical polymerization	•electromagnetic shield
[72]	•woven, 100% cotton, scoured, bleached, mercerized mass 130 g/m^2^, 1100 mm × 70 mm ^†^				•immersion in solution, pressed through rollers	•static protection and sensors for smart textiles
[73]	•plain weave, 100% cotton				•connected with gamma ray irradiation-induced grafting polymerization	•multifunctional fabric for harsh or sensitive conditions
**Poly (3,4-Ethylenedioxythiophene)**						
[74]	•woven, 100% polyester ^†^				•layer stack: silver, barium titanate, zinc oxide, poly(3,4-ethylenedioxy-thiophene) poly(styrenesulfonate) with screen printing; polyurethane/acrylate, silver, poly(3,4-ethylenedio-xythiophene) poly(styrenesulfonate)	•light-emitting device
[75]	•plain woven: five cotton; three linen; two silk; one wool gauze; one bamboo rayon fabric; two bast fiber (pineapple, banana)				•vapor deposition	•electrically conductive textile circuit components of smart textiles ^†^
[76]	•plain woven, 100% polyester mass 168 g/m^2^				•vapor-phase polymerization	•smart fabrics ^†^
[77]	•plain woven, 100% polyester 30 warp/10 mm, 22 weft/10 mm, mass 158 g/m^2^				•laboratory coating machine, dry, anneal vacuum and air condition; immersion, cure	•wireless communication for healthcare
[78]	•cloth fabric ^†^				•printed	•strain sensor, knee, wrist rehabilitation
**Carbon nanotubes**						
[79]	•woven, 100% cotton ^†^				•dipped in solution	•pressure sensor
[80]	•twill, 100% wool washed with nonionic detergent, 18 warp/10 mm, 16 weft/10 mm (cm)				•impregnating bath	•electrically conductive textile ^†^
[81]	•plain woven, 100% wool scoured, mass 67.6 g/m^2^, density 22.2 dtex of 36 filaments, fineness 21.5 µm, length 65 mm				•ultrasonic bath	•static dissipation, anti-spark, electromagnetic shielding, heating
[82]	•plain woven, 100% cotton pre-purified/washed, 29.5 warp/10 mm, 20.5 weft/10 mm (cm), mass 145 ± 7 g/m^2^, thickness 0.36 mm, yarn density 25 tex				•horizontal double-roll padding	•electrically conductive superhydrophobic fabric ^†^
[83]	•twill, 100% cotton mass 206.3 g/m^3^, thickness 0.41 mm, density 503.17 kg/m^3^				•screen printing with automatic squeegee, MS-300FRO	•chemical vapor sensor
[84]	•plain woven, 100% cotton				•dip coating, screen printing	•energy storage
[85]	•twill, 100% wool scoured, 16 warp/10 mm, 18 weft/10 mm (cm), mass 350 g/m^2^, fineness 30 Nm				•impregnating bath	•electrically conductive fabric ^†^
[86]	•woven, 100% cotton ^†^				•dipped in solution	•wearable electronic, energy storage
[41]	•plain woven, polyamide/Lycra^® †^				•coated	•stretch sensor
**Reduced Graphene Oxide**						
[87]	•plain woven, 100% poly (ethylene terephthalate) 39 warp/10 mm (390 threads/10 cm), 32 weft/10 mm (320 threads/10 cm), mass 89 g/m^2^, thickness 0.19 mm, warp 84 dtex f48, weft 150 dtex plain woven, 100% polypropylene 46 warp/10 mm (460 threads/10 cm), 33 weft/10 mm (330 threads/10 cm), mass 72 g/m^2^, thickness 0.19 mm, yarn 84 dtex f33				•roll padding in graphene oxide	•electrically conductive textile ^†^
[88]	•crepe de chine, 100% silk cleaned with sodium carbonate				•dip, dry, reduction	•medical care, electron device ^†^
[89]	•woven, 100% nylon, 100% cotton, 100% polyester ^†^				•immersion in graphene oxide, reduction	•electrooculography
[90]	•plain woven, 100% polyester, 70 g/m^2^				•immersion in graphene oxide, reduction and nanotitanium dioxide nucleation	•electroconductive, antistatic, UV protective fabric
[91]	•woven, 100% cotton, linen, viscose, polyester warp/10 mm 30, 24, 26, 40; weft/10 mm (cm) 22, 40, 24, 50; mass 94 g/m^2^, 67 g/m^2^, 141 g/m^2^, 68 g/m^2^; thickness 0.32 mm, 0.17 mm, 0.34 mm, 0.11 mm, respectively ^†^				•graphite and polyurethane coating applied with doctor’s knife	•electrically conductive fabric ^†^
[92]	•100% polyester ^†^				•ink jet printing	•wearable textile electronic circuits ^†^
[93]	•100% cotton 25.98 warp/10 mm (66 ends/inch), 22.83 weft/10 mm (58 picks/inch), mass 140 g/m^2^, thickness 0.41 mm, warp 28^s^Ne, weft 19^s^Ne ^†^				•immersion in graphene oxide, reduction	•electrically conductive fabric ^†^
[94]	•3/1 twill, 100% cotton				•continuous pad drying	•e-textiles ^†^
[95]	•plain woven, 100% polyester 43 yarns/10 mm, (110 yarns/inch), thickness 0.22 mm				•immersion in graphene oxide, reduction	•plantar pressure sensor, gait analysis
[96]	•woven, 100% cotton 12.99 warp/10 mm (33 warp/inch), 25.20 weft/10 mm (64 weft/inch), thickness 1 mm ^†^				•dipped in graphene nanoribbons (unzipped multiwalled carbon nanotubes)	•potential for strain sensor, conductive textiles
[97]	•100% cotton 40 × 40 yarn, 130 g/m^2 †^				•graphene oxide with vacuum filtration, thermally reduced	•strain sensor
[98]	•plain woven, 100% wool mass 170 g/m^2^				•immersion in graphene oxide and titanium dioxide, reduction	•electrically conductive textile ^†^
[99]	•cotton t-shirt nickel nitrate treated ^†^				•immersion in graphene oxide	•energy storage
[100]	•woven (plain based on image), 100% silk ^†^				•immersion in graphene oxide, reduction	•electrically conductive fabric ^†^
[101]	•woven, 100% polyester plasma treatment, 55 warp/10 mm, 29 weft/10 mm (cm), mass 100 g/m^2 †^				•immersion in graphene oxide, reduction	•electrically conductive fabric ^†^
[102]	•woven, 100% cotton (ISO 105/F standard fabric), 35 warp/10 mm, 31 weft/10 mm (cm), mass 115 g/m^2^				•immersion in graphene oxide, reduction	•counter electrode
[103]	•woven, 100% polyester 55 warp/10 mm, 29 weft/10 mm (cm), mass 100 g/m^2^				•immersion in graphene oxide and polypyrrole solution	•electrically conductive fabric ^†^
[104]	•plain woven, 100% polyamide				•immersion	•electrocardiogram
[105]	•plain woven, 100% wool mass 300 g/m^2^, worsted yarn plain woven, 100% cotton mass 141 g/m^2^				•graphene oxide painted on fabric, reduction	•e-textiles (e.g., glove to operate smart devices)
[106]	•plain woven, 100% cotton 30 warp/10 mm, 28 weft/10 mm (cm), mass 102 g/m^2^, 16.3 tex				•immersed in graphene oxide, titanium dioxide, reduction	•multifunctional fabric ^†^
[107,108]	•100% polyester 20 warp/10 mm, 60 weft/10 mm (cm), mass 140 g/m^2^, linear density warp 167 dtex, weft 500 dtex, fiber diameter 17 µm ^†^				•immersion in graphene oxide, reduction	•electrically conductive fabric ^†^
[109]	•plain woven, 100% cotton mass 190 g/m^2^				•pad-dry-cure of graphene nanoplate and waterborne anionic aliphatic polyurethane composite	•multifunctional fabric ^†^
[110]	•twill, 100% cotton thickness 300 mm, yarn thickness 100 mm *				•graphene oxide painted on the fabric, reduction	•supercapacitor
[111]	•woven, 100% polyarylate yarn 22.9 cN/dtex ^†^				•dyed in graphene oxide, reduction	•electrically conductive fabric ^†^
**Nickel**						
[112]	•plain woven, 100% cotton mass 182 g/m^2^				•immersion	•strain sensor
[113]	•woven textile fiber fabric, 100% polyester ^†^				•electroless plating of copper, nickel, silver and a layer of multiwalled carbon nanotubes	•supercapacitor
**Copper**						
[59]	•plain woven, 100% nylon polyurethane laminated or dry-coated 15 mm × 30 mm^2^				•sputtering copper, 2 µm	•motion sensor, electrodes
[59]	•ripstop (warp 50 d/36 f, weft 75 d/54 f, 0.12 mm, 111.8 g/m^2^) and mesh (monofilament 50 µm, 0.08 mm, 30.0 g/m^2^)				•electroless plating of copper and nickel (2 µm)	•motion sensor, electrodes
**Silver**						
[114]	fiber content (100%) ^†^	yarn/10 mm (thread/10^−1^ m)		yarn (tex)	•screen printing, sputtering	•circuit including capacitor sensor input, controller system-on-a-chip, light emitting diode
	cotton viscose silk wool polyester	21.1 47.9 49.3 31.2 21.0 30.0 50.0	211 479 493 312 210 300 500	20.0 21.0 8.0 43.0 5.5 5.5 4.4		
[115]	•plain weave, 100% cotton 160 g/m^2^				•immersion	•hygienic jacket for X-ray use, electromagnetic shielding
**Knit**						
**Constructed in Fabric**						
**Stainless Steel**						
[40]	•knit ^†^				•316 L stainless-steel fiber in yarns; polypyrrole, manganese dioxide, reduced graphene oxide	•energy storage
[116]	•warp knit net, tetra-channel polyester				•metal clad aramid fibers for signal conductors	•thermal survival smart clothing for Arctic environment
[117]	•warp knit				•stainless-steel wire, 150d/144f antibacterial nylon, 75d/48f crisscross-section polyester filaments as core, Z-direction cover, and S-direction cover, respectively	•electromagnetic shield
[59]	•single jersey, 50% cotton/50% stainless steel 11.44 wales/10 mm, 9.8 courses/10 mm (57 wales × 49 courses per 5 cm/5 cm), mass 155.0 g/m^2^, thickness 0.44 mm				•stainless steel	•motion sensor, electrodes
[41]	•1 × 1 rib, polyamide/polyester ^†^				•Bekintex 50/2; Shieldtex^®^ 235/1 × 2	•stretch sensor
[118]	•single jersey, 100% wool knee garment				•stainless steel; silver-coated nylon; Europa gill 40% polyester/40% copper sulfides/20% stainless steel	•strain sensor
[49]	•plain knit; wave knit *				•Beakart Bekinox^®^VN (500 tex/f275/2) 100% stainless-steel yarn; Beakart Bekitex BK 50/2 (40 tex), 20% stainless steel/80% polyester	•electrical electrodes to monitor heart rate
[119]	•intarsia knit, Meryl^®^ Skinlife fiber produced by Nylstar base, Lycra^®^				•electrodes: Bekinox^®^ VS stretch-broken sliver 100% stainless steel; Belltron^®^ 9R1 polyamide core, carbon cover; conductive elastomer: silicon rubber graphite mixture	•vital parameters, movement ^†^
[120]	•tubular intarsia knit, 100% viscose				•two stainless-steel wires twisted around viscose yarn	•cardiovascular diseases
[121]	•’Textrodes’ ^†^				•stainless-steel electrodes	•monitoring suit ^†^
**Silver**						
[122]	•single jersey, 100% Nomex^®^				•silver-coated polyamide (Shieldtex^®^ 234/34-2 ply HC, 234 dtex, 32 filaments), encapsulated in thermoplastic polyurethane film	•potential sensors, actuators, power, microprocessors, data transmission,
[123]	•knit ^†^				•Shieldtex^®^ MedTex P130, cured in Ecoflex^®^ 30 (silicone); interdigital sensor area carved with burning	•strain sensor
[124]	•core spun Lycra^®^ 800 dtex, 570 dtex, 156 dtex with nylon core, three variations of knit				•silver-coated nylon	•strain sensor
**Polymers**						
[116]	•warp knit net, tetra-channel polyester				•carbon conductive weave for temperature sensors/control	•thermal survival smart clothing for Arctic environment
[125]	•crochet knit *				•carbon-coated fiber (RESISTAT F901) single and double wrapped around rubber (Φ0.5 mm) and polyester (333 dtex) core	•piezoresistive sensor
[126]	•knit, 100% chlorine-Hercosett^®^ (Hercules) merino wool 1.3 mm tex, treated fibers in slivers, spun in yarn ^†^				•polypyrrole-treated fibers	•apparel for static dissipation, anti-spark, electromagneticinterference shielding
[127]	•knit, 100% wool ^†^				•polypyrrole chemical oxidative polymerization of fibers	•electrically conductive fabric ^†^
[128]	•single-, double-, four-ply knit of Spandex (40 denier) or polyester (100 denier)				•polyurethane/poly (3,4-ethylenedioxythiophene): poly(styrenesulfonate) fibers	•strain sensor
[129,130]	•plain-knit, co-knit, co-knit alternative, co-knit with conductive stitch, plain knit with nonconductive stitch; polyester (70 denier/50 filament) for co-knit				•polyurethane/poly (3,4-ethylenedioxythiophene): poly(styrenesulfonate) multifilaments	•strain sensor
**Carbon nanotubes**						
[131]	•three-layered weft-knit 20 courses/10 mm 12 wales/10 mm (cm); different inner, middle, outer layer, and yarn content (details provided in paper)				•polyester yarn with carbon core; 80% polyester/20% stainless steel	•multifunctional wearable fabric ^†^
[132]	•knit with flat-bed machine, two rows separated by two rows of nonconductive yarns				•activated carbon in swelled cellulose yarn of linen, bamboo, viscose (cotton could not be knitted)	•supercapacitor
[133]	•’ordinary textile’, 100% cotton (image appears to be woven) ^†^				•carbon nanotubes treated yarn	•ammonia sensor
[134]	•knit, 75% electro-conductive yarn/25% Lycra^® †^				•Belltron^®^	•piezoresistive sensor
**Graphene**						
[135]	•interlock, 100% cotton				•yarns batched dyed followed by integration in interlock structure	•temperature sensor
**Not described**						
[136]	•knit strips (10 mm wide, 10 m length); knit conductive tracking *				•conductive threads	•context awareness ^†^
**Chemical treatment**						
**Polypyrrole**						
[137]	•interlock, 100% polyester mass 106 g/m^2^ (100 mm × 50 mm)				•vapor polymerization	•electrically conductive fabric ^†^
**Reduced graphene oxide**						
[138]	•100% cotton (image shows knit) ^†^				•immersion in graphene oxide, reduction, immersion in single-walled carbon nanotubes	•motion sensor
[139]	•weft knit, 90% nylon/10% spandex 12 wales/10 mm, 26 courses/10 mm (cm), mass 99.5 g/m^2^				•immersion in graphene oxide, reduction	•strain sensor
[140]	•100% polyester (diagram shows knit) ^†^				•immersion in reduced graphene oxide, variation with polypyrrole	•energy storage
[141,142]	•weft knit, 100% cotton mass ≈220 g/m^2^, thickness 0.55 ± 0.05 mm, yarn diameter ≈223.9 ± 27.4 μm, measured fiber diameter ≈15.1 ± 0.8 μm weft knit, 100% wool, mass ≈380 g/m^2^, thickness ≈0.7 ± 0.06 mm, yarn diameter ≈509.7 ± 34.1 μm, fiber diameter ≈49.8 ± 4.6 μm				•immersion in ultrasonication bath	•strain sensor
[143]	•interlock, 100% polyamide				•immersion, reduction	•sensitive artificial skin
[93]	•knit, 100% cotton, 12.60 wales/10 mm (32 wales/inch), 22.83 courses/10 mm (58 courses/inch), mass 140 g/m^2^, thickness 0.58 mm, yarn count 30^s^Ne, loop length 2.72 cm				•immersion in graphene oxide followed by reduction reaction	•electrically conductive fabric ^†^
[144]	•knit, 100% polyester ammonia treated mass 100 g/m^2 †^				•immersion in graphene oxide, reduction, variation with silver nanoparticles with immersion	•may be used for supercapacitors, sensors, solar cells
[145]	•knit, 100% viscose 21 wales/10 mm, 15 courses/10 mm (109 wales/5 cm, 75 course/5 cm) ^†^				•multicycle dipping‒drying graphene oxide, reduction	•potential energy storage
[146]	•plain knit, 100% cotton *				•spray coating layer by layer assembly	•multifunctional (UV protection, electrical conductivity, electromagnetic shielding)
[147]	•knit, 100% nylon Lycra^® †^				•immersion in graphene oxide, reduction, polypyrrole polymerization	•supercapacitor
**Nonwoven**						
**Silver**						
[148,149,150]	•Freudenberg’s Evolon^®^ (polyester and nylon); BBA FiberWeb’s Resolution Print Media (trilobal polyester); DuPont’s Tyvek^®^ (polyethylene)				•screen printed	•flexible circuit boards
**Polyaniline**						
[151]	•polypropylene ^†^				•immersion	•gas sensor
**Reduced graphene oxide**						
[152]	•polyester mass 40 g/m^2^				•suction filtration with graphene oxide dispersion, reduction	•multifunctional wearable smart device ^†^
[153]	•nonwoven ^†^				•immersion and reduction	•strain sensor
[154]	•poly (ethyleneterephthalate) simulated leather ^†^				•immersion and sonication	•smart textile ^†^
[155]	•mat, aramid (Kevlar^®^), polyester, cotton, nylon ^†^				•layering	• wearable electronic devices ^†^, energy harvesting
[156]	•poly (ethylene terephthalate) ^†^				•dyed	•heating elements
[157]	•nonwoven ^†^				•immersion in graphene oxide, reduction; some additionally immersion in polypyrrole and multiwalled carbon nanotubes	•capacitor
[158]	•nylon-6 ^†^				•electrospinning and wrapped around nylon nanofibers in plane of random orientation	•conductive wires and functional fabrics in wearable electronics ^†^
**Fabric structure not described**						
**Silver**						
[159]	•commercially available tight fitting stretch T-shirt, 100% polyamide ^†^				•zig-zag embroidered	•electrocardiogram
**Nickel**						
[160]	•100% silk ^†^				•electroless plating	•humidity sensor
**Gold**						
[161]	•100% cotton, silk, wool, polyester; 60% cotton/40% polyester, 60% wool/40% polyester, 50% wool/50% viscose, 10% wool/90%viscose ^†^				•droplet deposition, immersion	•chemical sensor
**Poly (3,4-ethylenedioxythiophene)**						
[162]	•polyamide/Lycra^®^ density 63 g/m^2^; thickness 294 µm ^†^				•immersion	•electrocardiogram
[163]	•100% polyester ^†^				•spray coating	•capacitor, heating/electronic devices
**Polypyrrole**						
[137]	•scoured and bleached cotton ^†^				•electrochemical and chemical polymerization	•electrically conductive fabric ^†^
**Reduced graphene oxide**						
[164]	•piece of jeans, polyester from lab coat, nonwoven material from swabs *				•spray nozzle under pressure	•strain sensor
[142]	•cotton cloth mass 220 g/m^2^, yarn diameter 223.9 ± 27.4 µm, fiber diameter 15.1 ± 0.8 µm ^†^				•immersion, stirring	•strain sensor
[165]	•polyester ^†^				•graphene film encapsulated with insulating glue	•electrocardiogram
[166]	•100% cotton ^†^				•immersion in graphene oxide followed by reduction	•smart and e-textiles ^†^
[167]	•100% cotton ^†^				•immersion in graphene oxide followed by reduction	•multifunctional, electroconductive, superhydrophobic ^†^
[168]	•100% cotton mass 114 g/m^2 †^				•immersion in graphene oxide followed by reduction, chemical polymerization of polypyrrole	•supercapacitor
[169]	•commercial cotton ^†^				•immersion followed by reduction	•capacitor

**Table 2:** Standard methods to determine the electrical properties of apparel fabrics and yarns.

Title, Reference, Use	Scope
•ASTM D4496:2013 Standard test method for D-C resistance or conductance of moderately conductive materials [185]	•suitable for materials composed of conductive and resistive components, not considered good insulators or conductors, volume resistivity 10^0^ Ω/cm to 10^7^7 Ω/cm or surface resistivity 10^3^ Ω/square to 10^7^7 Ω/square •often anisotropic so is dependent on orientation •standard conditions 23 °C and 50% RH, but can be measured in other conditions
•ASTM D257-07 Standard test methods for DC resistance or conductance of insulating materials [93,115,186]	•volume and surface resistivity of insulating materials greater than 10^7^ Ω/cm or 10^7^ Ω/square •volume/surface resistivity cell and megohmmeter •fabric between two electrodes in cell, measure electrical-resistance with megohmmeter at applied voltage after 60 s •surface resistance calculated with a formula
•AATCC 76:2018 Electrical resistivity of fabrics (2011, 1995 is an earlier version) applicable to resistivity greater than 10^7^ Ω/cm or 10^7^Ω/square [61,93,154,184,187]	•how surface resistance affects electrostatic dissipation of fabric •surface resistivity cell and megohmmeter •fabric between two electrodes in cell, measure electrical resistance with megohmmeter at applied voltage after 60 s •surface resistance and conductivity calculated with a formula •same set up can be used to measure properties in accordance with ASTM D257-07 Standard Test Methods for DC Resistance or Conductance of Insulating Materials
•EN-BS 16812:2016 Textiles and textile products, electrically-conductive textiles, determination of the linear electrical resistance of conductive tracks [183]	•linear resistance of conductive tracks for textile structures
•BS EN 1149-1:2006 Protective clothing. Electrostatic properties. Test method for measurement of surface resistivity; BS EN 1149-2:1997 Protective clothing. Electrostatic properties. Test method for measurement of the electrical resistance through a material (vertical resistance) [131,188,189]	•measures surface resistance •quantify electrostatic dissipation and prevent discharge
•Standard Recommendation S.R. CEN/TR 16298:2011 textiles and textile products; smart textiles; definitions, categorization, applications and standardization needs [190]	•provides advice and information for consideration when writing standards for smart textiles •expertise from multiple disciplines is required: textiles, medical devices, electronic devices •tests to be suitable for the textile components and electronic components •synergies from combining textiles and electronic components

**Table 3:** Thermal and moisture transfer of functionalized fabrics.

Materials	Key Results	Reference
•woven, 100% cotton, 25.98 warp/cm, 22.83 weft/cm, warp 28^s^Ne, weft 19^s^Ne, mass 140 g/m^2^, thickness 0.41 mm •knit, 100% cotton, 22.83 course/cm, 12.60 wales/cm, yarn count 30^s^Ne, loop length 2.72 cm, mass 140 g/m^2^, thickness 0.58 mm •graphene oxide treated, chemically reduced	•knit had higher add on (3.95% > 3.31%) due to porosity, bulk •electrical resistivity of knit 0.19 MΩ/square < woven 0.26 MΩ/square •air permeability decreased 41% for knit, 27% for woven •pore size decrease, thickness increase for both •water vapor permeability decreased: 2287 g/m^2^/day, 2000 g/m^2^/day to 1740 g/m^2^/day, 1700 g/m^2^/day for knit and woven, respectively	[93]
•plain woven, 100% cotton, 160 g/m^2^ •silver nanoparticle treatment, 50, 100, or 150 dips (30 second immersion, dried 100 °C 3 min), binder added	•decreased electrical resistivity with coating, partially covers pores of fabrics •negligible decrease in air permeability, i.e., 790 mm/s > 782 mm/s, 770 mm/s, 756 mm/s after 50, 100, 150 dips, respectively •small change in water vapor permeability 78.8% > 77.5%, 74.6%, 73.9% 50, 100, 150 dips, respectively	[115]
•three-layered weft knit, 67% cotton/33% polyester, carbon core polyester filament, and hollow polyester yarns with polypropylene yarns or 80% polyester/20% stainless-steel yarns •micro porous polyurethane treatment	•increased mass, thickness with treatment •high decrease in permeability to air and water retentivity •comparatively lower decrease in permeability to water vapor •surface wetting increased with coating (from grade one to grade three) •resistance to water penetration increased with coating	[131]
•twill, plain woven, 100% wool •polypyrrole and carbon treatment	•increased thermal conductivity following polypyrrole treatment; minimal change in thermal conductivity following carbon sputter coating	[65]
•woven, 100% polyester with light-emitting devices	•air permeability increased with pixel size of light-emitting devices	[74]
•warp knit, stainless-steel wire, 150d/144f antibacterial nylon, 75d/48f crisscross-section polyester filaments as core, Z-direction cover, and S-direction cover, respectively	•air permeability increased with increased polyester content, greater than 40 cm^3^/cm^2^/s	[117]

**Table 4:** Research papers investigating the durability to washing of functionalized fabrics.

Wash Type Reference	Fabric Structure and Fiber Content	Method	Result
**Standard method**			
**International**			
[45]	•100% silk integrated with copper polyamide substrate connected to touch sensor encapsulated with polydimethylsiloxane	•ISO 6330:2012; type A washing machine, 2 kg cotton fabric: 15 min 30 °C 1200 rpm, 37 min 30 °C 400 rpm, 42 min 30 °C 400 rpm with fabric conditioner (35 mL) and detergent (37 mL); dried flat on a stainless-steel rack at 25 °C for 90 min	•bending and twisting; maximum charge voltage decreased as the number of washes increased; circuit function lost after 1 wash for 800 rpm, retained 10 to 15 washes with wash of 400 rpm
[122]	•100% Nomex^®^ single jersey with silver-coated polyamide covered with a thermoplastic polyurethane film	•ISO 6330:2012; 40 °C for 30 min with a Datacolour Ahiba IR laboratory dying machine for 50 consecutive washes	•increased electrical resistance of approximately four times was reported after 50 washes
[77]	•plain woven, polyester 158 g/m^2^, 30 warp/10 mm, 22 weft/10 mm coated with poly(3,4-ethylenedioxythiophene) polystyrene sulfonate for 5 min	•ISO 6330:2012; type A washing machine, 100% polyester ballast	•increased surface resistance following each wash; following 10 washes increased magnitude of 100
[115]	•plain woven, 100% cotton, 160 g/m^2^ treated with silver nanoparticles	•ISO 105 C10: 2006A; 5 g/L standard detergent, liquor ratio 50:1, samples rinsed 30 min at 40 °C, dried at 25 °C 65%RH	•no significant decrease in electrical conductivity after washing (with binder over silver coating)
[93]	•woven, 100% cotton, 25.98 warp/cm, 22.83 weft/cm, warp 28^s^Ne, weft 19^s^Ne, mass 140 g/m^2^, thickness 0.41 mm; knit, 100% cotton, 22.83course/cm, 12.60 wales/cm, yarn count 30^s^Ne, loop length 2.72 cm, mass 140 g/m^2^, thickness 0.58 mm; dipped in graphene oxide, chemically reduced	•ISO 105 C10:2006A; 5 g/L soap at 40 °C, for 30 min	•surface electrical resistivity increased following washing: 0.19 MΩ/square and 0.26 MΩ/square to 1.75 MΩ/square and 2.39 MΩ/square for knit and woven, respectively
[94]	•3/1 twill, 100% cotton treated with reduced graphene oxide	•ISO BS EN 105 C06; 4 g/L reference detergent, 10 stainless-steel balls at 40 °C for 30 min	•electrical resistance increased from 36.94 KΩ/square to 70.32 KΩ/square after first wash; 139.09 KΩ/square after 10 washes
[146]	•plain knit, 100% cotton with spray coating layer by layer of graphene solution	•ISO 105-C03	•surface resistance increased after wash
[126]	•polypyrrole-treated wool fibers spun in yarn (36 tex) and knitted	•ISO BS EN 105 C06; EN ISO 105-X05:1997; Original Hanau Linitest apparatus, ECE detergent and tetrachloroethene extra pure, respectively	•after three wash cycles increase of 11.3ρs and 44.8ρs resistivity, color degradation; after three organic solvent washes increase 1.02ρs and 1.06ρs resistivity, no color degradation
[127]	•100% wool polypyrrole treated	•EN ISO 105-C06:1997 A1S; EN ISO 105-X05:1997; Original Hanau Linitest apparatus using ECE detergent and tetrachloroethene extra pure, respectively	•exponential decrease in electrical conductivity was observed following domestic and commercial washing
[116]	•circuit boards and cables •temperature sensors, sound •circuit boards	•ISO 6330 15 times, 40 °C, 60 °C •ISO 6330 10 times 40 °C •dry cleaning with perchlorethylene	•remained operational after all wash and dry cleaning processes
**United States of America**			
[90]	•plain woven, 100% polyester, 70 g/m^2^, treated with reduced graphene oxide	•AATCC 61-2A, 50 °C for 30 min, liquor ratio 1:50 with AATCC soap, 5 cycles	•surface and volume resistivity increased
[109]	•plain woven, 100% cotton, mass 190 g/m^2^ treated with graphene nanoplate and polyurethane dispersion	•AATCC 61-2006; 500 mL (75 mm × 125 mm) stainless-steel lever lock canisters; 200 mL standard detergent with 10 stainless-steel balls	•surface electrical resistivity increased from 2.94 × 101^−1^Ω/m to 3.35 x101^−1^Ω/m after 10 washes
[73]	•100% cotton treated with polyaniline	•AATCC 132:2004; AATCC 86:2005; in capped bottles with 200 mL TTE detergent solution at 30 ± 2 °C for 30 min, intense stirring, 40 washes	•surface resistance was stable after 40 washes
[76]	•plain woven, 100% polyester mass 168 g/m^2^ treated with poly(3,4-ethylenedioxythio-phene) with and without fluorinated decyl polyhedral oligomeric silsesquioxane	•Australian Standard (AS 2001.1.4), 5 cycles	•surface resistance increased with increased cycles from 1.0 KΩ/square to 1.9 KΩ/square and 0.6 KΩ/square to 2.3 KΩ/square with and without the additive
**Other**			
[162]	•polyamide/elastane poly(3,4-ethylenedioxythiophene) polystyrene sulfonate coated	•commercial detergent (X.TRA Total, France) in domestic laundering machine (Miele, France); 35 min at 40 °C with 30 mL detergent, total machine load 2.5 kg, 600 rpm; corresponding to ISO 6330	•after 50 wash cycles the power spectral density decreased for one solution, while the other showed minimal differences
[50]	• woven knitting wool with nickel/titanium filament covered with polyurethane filaments	•dipped in detergent dissolved in water, thoroughly rubbed with the hand, rinsed with water, naturally dried	•maintained the same signal level
[138]	•100% cotton (image show knit) coated with reduced graphene oxide and single-walled carbon nanotubes	•rinsing in deionized water with a magnetic stirrer, 10 kPa pressure, 10 cycles	•minimal change in resistance
[91]	•woven 100% cotton, 100% viscose, 100% linen, 100% polyester coated with graphite/polyurethane dispersion	•household washing machine, heavy duty detergent at 40 °C, 1400 rpm, 10 or 50 cycles	•graphite flakes removed after 10 cycles •electrical resistance increase greatest for viscose and polyester •less change observed with higher graphite concentration and fine flakes
[164]	•piece of jeans, polyester from lab coat, nonwoven material from swabs graphene-treated	•beaker with water, 450 rpm for 16 h	•no delamination
[97]	•100% cotton 40 × 40 yarn, 130 g/m^2^, treated with graphene oxide with vacuum filtration, thermally reduced	•Labortex oscillating type dyeing machine, 100 mL deionized water, 2 mg/mL sodium carbonate, 5 mg/mL soap, 60 °C for 30 min, 10 cycles	•0.9 KΩ/square before wash, remained lower than 2 KΩ/square after 10 washes
[92]	•100% polyethylene terephthalate inkjet printed with graphene with a polyurethane layer	•immersion in 100 mL deionized water with 2 mg/mL sodium carbonate and 5 mg/mL soap at 50 °C in a beaker, tumble washed for 30 min according to Ren et al. [97]	•decreased performance, but still electrically conductive after 20 cycles
[75]	•100% silk poly(3,4-ethylenedioxythiophene) polystyrene sulfonate coated	•’vigorously stirred, commercial laundry detergent, 10 min, rinsed in water	•the coating was not largely effected by laundry detergent or mechanical stress
[98]	•plain woven 100% wool treated with graphene/titanium dioxide	•60 °C for 20 min, 1 g/L nonionic detergent, rinsed with distilled water, dried	•durable to wash based on minimal change in electrical resistivity after one wash
[47]	•woven, 100% polyamide filament coated with copper in warp and weft, 52 ± 5 g/m^2^	•perchlorethylene in a two-bath procedure, 16 kg load for 10 cycles (both 20 °C, duration 4 min and 6 min, 300 rpm and 360 rpm, bath ratio 1:2 and 1:4, respectively, detergent mega super star in bath one only. Dried 60 °C for 30 min, ironed at 110 °C following suppliers instructions after each wash	•electromagnetic shielding effectiveness decreased following increased dry cleaning cycles •visible degradation was apparent from scanning electron microscope, increasing with increased cycles, ironing also had a noted effect on degradation, i.e., the coating was not continuous
[96]	•woven, 100% cotton treated with multiwalled carbon nanotubes	•immersion in 100 mL water at 40 °C, stirred 600 r/min for 20 min, dried 60 °C, 10 repeats	•after ~5 washes electrical resistance stabilized
[86]	•woven, 100% cotton	•washed in water (soaked, squeezed, wrung out)	•’outstanding performance’
[120]	•WEALTHY	•rinsed with water, Marsilia soap	•considered washable

## Supplementary Materials

The following are available online at https://www.mdpi.com/1424-8220/19/16/3570/s1, Table S1: Terms and definitions facilitate understanding for a multidisciplinary readership; Table S2: Standard methods for determining properties of fabrics: examples of international and national standards to determine fabric properties.
