# Supplementary material for: Fabrics and Garments as Sensors: A Research Update
Source: Sensors (Basel). 2019 Aug 15;19(16):3570. doi: 10.3390/s19163570 (PMC6719058; doi:10.3390/s19163570)
Supplement: Supplementary file 1 [file sensors-19-03570-s001.pdf]

sensors

MDPI

Table S1. Terms and definitions.

Term, reference	Definition
•apparel [1]	•personal outfit, garments, clothing or attire, including headwear and footwear; clothing more specific to those covering the body or limbs.
•coated fabric [1]	•1 a material composed of two or more layers, at least one of which is a textile fabric and at least one of which is a substantially continuous polymeric layer. The layers are bonded closely together by means of an added adhesive or by the adhesive properties of one or more of the component layers.<br>2 a textile fabric on which there has been formed *in situ*, on one or both surfaces, a layer, or layers, of adherent coating material.
•course (knit) [1]	•a row of loops (i) across the width of a flat fabric, or (ii) around the circumference of a circular fabric.
•durability [1]	•the ability of a textile to perform its required function until an agreed limiting state is reached.
•electrical resistance/ resistivity [1]	•inverse of electrical conductivity i.e. to resist electrical charge. Resistance is a measure of ohms, resistivity is ohms per length (e.g., millimeters, centimeters, meters); inverse is electrical conductiviy i.e. ability to transfer electrical charge.
•electronic textiles [2,3]	•a subset of smart textiles/apparel containing electronic components integrated in the structure (become part of the garment or other textile based product e.g. bedding or upholstery), which sense changes in the environment or the body, and can often respond in a particular way.
•fabric [1]	•a manufactured assembly of fibers and/or yarns that has substantial surface area in relation to its thickness and sufficient cohesion to give the assembly useful mechanical strength.
•fiber [1]	•textile raw material, generally characterized by flexibility, fineness and high ratio of length to thickness.
•filament [1]	•a fiber of indefinite length.
•flexural rigidity [1]	•a measure of the resistance of materials to bending by external forces. It is related to stiffness and is one of the factors sensed when a fabric is handled.
•intarsia knit [1]	•weft-knitted plain, rib, or purl fabrics containing designs in two or more colors (or textures) within the same course in which each area of color is knitted from a separate yarn, which is contained entirely within that area.
•knit fabric [1]	•fabric structure consisting of interlocking loops.
•man-made fiber [1]	•a fiber that does not occur in nature, although the material of which it is composed may occur naturally,' includes synthetic fibres 'made from a polymer

	synthesised from chemical elements or compounds, includes polyester, polyamide/nylon, acrylic, regenerated celluose (e.g., viscose).
•natural fiber [1]	•a fiber occurring in nature, includes wool, cotton, silk, linen.
•nonwoven fabric [1]	•fabrics normally made from continuous filaments or from staple fiber webs or batts strengthened by bonding using various techniques: these include adhesive bonding, mechanical interlocking by needling or fluid jet entanglement, thermal bonding and stitch bonding.
•plain weave [1]	•the simplest of all weave interlacings in which the odd warp threads operate over one and under one weft thread throughout the fabric with the even warp threads reversing this order to under one, over one, throughout.
•smart/intelligent textiles [4]	•the addition of something not typical of apparel (often electronic) providing additional functions but does not detrimentally alter the characteristics of the original textile/apparel e.g. wear, durability.
•staple fiber [1]	•a fiber of limited and relatively short length.
•textile [1]	•broad term, includes fibers, yarns, fabrics, apparel.
•twill weave [1]	•a weave that repeats on three or more ends and picks and produces diagonal lines on the face of the fabric.
•wales [1]	•a column of loops along the length of fabric.
•warp [1]	•threads lengthways in a fabric as woven.
•wearable technologies [4]	•items (often with electronic capabilities) worn on the body consisting of a simple interface performing tasks to satisfy needs of a specific target group, either worn as an accessory (wrist watch, hearing aids), implant (more permanent, invasive), or incorporated in textiles/apparel (smart textiles, e-textiles).
•weave/woven fabric [1]	•a pattern of interlacing of warp and weft in a woven fabric.
•weft [1]	•threads width ways in a fabric as woven.
•yarn [1]	•a product of substantial length and relatively small cross-section consisting of fibers and/or filament(s) with or without twist.

Table S2. Standard methods for determining properties of fabrics

**Title, reference, use in papers**	**Scope**
**Thermal and moisture transfer**	
•ISO 9237:1995 Textiles - Determination of the permeability of fabrics to air [5-8]	•air is forced through fabric specimens to determine volume of air passing through

•ISO 11092:2014 Textiles - Physiological effects - Measurement of thermal and water-vapor resistance under steady-state conditions (sweating guarded-hotplate test) [7,9]	•fabrics, films, coatings, foams, leather in single layer or multilayer assemblies for use of apparel, quilts, sleeping bags, upholstery can be tested<br>thermal and water-vapor resistance determined by temperature difference between the two material faces divided by heat flux per unit area in direction of the gradient
•ISO 4920:2012 Textile fabrics -- Determination of resistance to surface wetting (spray test) [8,10]	•test fabrics with water-resistance or water-repellent finishes, or those not treated but not rain resistance because penetration through fabric is not measured<br>distilled or deionized water of a specified volume is sprayed on a fabric at a 45° angle a specified distance from the spray nozzle<br>the fabric is positioned so the warp/wale/length direction is parallel to water flow<br>specimen appearance compared to descriptive standards and photographs
•ASTM D6767-16:2016 Standard Test Method for Pore Size Characteristics of Geotextiles by Capillary Flow Test [6,11]	•pore size ranging from 1 μm to 1000 μm; >500 μm accuracy may varies with apparatus<br>determined with surface analysis of specified pore size
•BS 7209:1990 Specification for water vapor permeable apparel fabrics [12]	•specifies requirements for water resistant clothing and permeable to water vapor<br>apparatus consists of shallow dishes on a rotating stage<br>fabric is sealed over the dish containing water and rotated for a minimum of 6 h<br>the mass difference is used to calculate water vapor permeability index
•BS EN 12127:1998 Textiles - Fabrics - Determination of mass per unit area using small samples [13]	•dry mass of the fabric is determined, then in conditions (e.g. 20 ± 2 ℃, 65 ± 4%RH) to return to a normal state; the difference is calculated as percent regain
•BS EN 20811:1992 Textiles. Determination of resistance to water penetration. Hydrostatic pressure test[8,14]	•distilled water is applied to one face of fabric specimens with a steady increase of pressure until penetration occurs at three places, pressure of third penetration is noted
**Elasticity (extension and recovery)**	
•ISO 13934-1:2013 Textiles - Tensile properties of fabrics - Part 1: Determination of maximum force and elongation at maximum force using the strip method [15]	•extension of a constant rate of fabric specimens of specified dimensions until rupture<br>maximum force and elongation at maximum force, force at rupture and elongation at rupture are measured
•BS EN 14704-1:2005 Determination of the elasticity of fabrics – Part 1: Strip tests [16]	•fabric strips in straight or loop form put under strain with a tensile tester<br>previously BS 4294:1968 Stretch and recovery properties of fabrics [17] and BS 45-2:1992 Methods of test for elastic fabrics [18]
**Wrinkle recovery**	

•ISO 9867:2009 Textiles - Evaluation of the wrinkle recovery of fabric - Appearance method [19]	•fabric specimens are wrinkled in a wrinkle device with a specified load for a specified time; hung for 24 h in a conditioned atmosphere<br>specimen appearance is compared to three-dimensional wrinkle recovery replicas
•ISO 7854:1995 Rubber or plastics-coated fabrics - Determination of resistance to damage by flexing [20]	•a flex testing machine is used where a folded strip of fabric is mounted between two flat grips<br>the specimen is bent at a rate of five times per second for a pre-set number of cycles or when deterioration occurs (e.g. cracking)
**Flexibility/stiffness**	
•ASTM D1388-14e1:2014 Standard test method for stiffness of fabrics [21]	•cantilever test, fabric bends under its own mass; heart loop test, fabric strip is in a loop and hung vertically<br>measure bending length, calculate flexural rigidity
•BS 3356:1990 Method for determination of bending length and flexural rigidity of fabrics [22]	•rectangular fabric specimens placed on horizontal platform, pushed in warp/wales/length direction to hang over the edge under own mass to angle 41.5°<br>flexural rigidity is determined from bending length and mass per unit area
•BS 5058:1973 Method for the assessment of drape of fabrics [23]	•a drapemeter is used to cast a shadow of the fabric draped over a surface<br>coefficient of drape is calculated from the mass of the initial specimen and that after cutting away the outer edge of the cast shadow
**Pilling and abrasion**	
•ISO 12945-2:2000 Textiles - Determination of fabric propensity to surface fuzzing and to pilling - Part 2: Modified Martindale method [24]	•fabric specimens are rubbed in a lissajous figure for specified intervals of the number of cycles: 125, 500, 1000, 2000, 5000, 7000<br>specimen appearance is compared to descriptive standards or photographs
•ISO 12947-2:2016 Textiles - Determination of the abrasion resistance of fabrics by the Martindale method - Part 2: Determination of specimen breakdown [25]	•fabric specimens are rubbed in a lissajous figure for specified cycles of intervals until specimen breakdown i.e. yarn breakage
•ISO 105-X12:2016 Textiles -- Tests for color fastness -- Part X12: Color fastness to rubbing [6,26]	•a dry or wet rubbing cloth can be used<br>straight line rubbing motion, two alternative size of rubbing fingers, one for pile fabrics and one for those with solid color/print
**Wash and dimensional stability**	
•ISO 3759:2011 Textiles – Preparation, marking and measuring of fabric specimens and garments for determination of dimensional change [27]	•specifies marking squares of fabric to measure dimensional change after wash

•ISO 6330:2012 Textiles - Domestic washing and drying procedures for textile testing [28,29]	•fabric specimens washed in automatic washing machine and dried<br>thirteen washing procedures for horizontal axis front loading machine<br>eleven procedures for vertical axis, top loading agitator type<br>seven procedures for vertical axis, top loading pulsator top<br>six drying procedures: line dry, drip line dry, flat dry, drip flat dry, flat press, tumble dry; six detergents specified<br>ballasts are used to make up the weight of wash
•ISO 105 C10:2006A Textiles -- Tests for color fastness -- Part C10: Color fastness to washing with soap or soap and soda [6,7,30]	•effect of washing on color fastness<br>fabric specimen is contacted with one or two fabrics is mechanically agitated for a specific time, temperature, typically in water bath with stainless steel containers rotated containing stainless steel balls<br>multifiber fabric or two single fiber fabrics, soap, or soap and soda solution used<br>rinsed and dried<br>colorfastness determined with grey scale or instrumentally
•ISO BS EN 105 C06 Textiles – Tests for color fastness - Part C06: Color fastness to domestic and commercial laundering [31-33]	•color fastness to domestic and commercial laundering used for normal household textiles with a reference detergent<br>fabric specimen is contacted with one or two fabrics is mechanically agitated for a specific time and temperature, typically in water bath with stainless steel containers rotated containing stainless steel balls<br>multifiber fabric or two single fiber fabrics
•AATCC 132: 2004 Color fastness to drycleaning [34,35]	•determine color fastness of textiles to dry cleaning, commercial<br>not appropriate for evaluating durability of textile finishes or resistance of color to spot/stain removal
•AATCC 86: 2016 Drycleaning: Durability of applied designs and finishes [35,36]	•effect of multiple drycleaning cycles of textiles with applied designs or finishes<br>determining resistance of colors to spot and stain removal with drycleaning<br>not appropriate to test color fastness to drycleaning<br>specimens agitated in solution of solvent, drycleaning detergent with steel balls
•AATCC Test method 61-2013 Colorfastness to Laundering: Accelerated [37,38]	•determine colorfastness to laundering of textiles<br>five typical hand or home wash, with or without chlorine, approximately one 45 min test

Reference

1. The Textile Institute. Textile Terms and Definitions. Available online: www.ttandd.org (accessed on 21 March 2019).

2. Stoppa, M.; Chiolerio, A. Testing and evaluation of wearable electronic textiles and assessment thereof. In *Performance Testing of Textiles: Methods, Technology and Applications*, Wang, L., Ed. Woodhead Publishing: Cambridge, England, 2016; pp. 65–101, doi:10.1016/B978-0-08-100570-5.00005-0.
3. Ghahremani Honarvar, M.; Latifi, M. Overview of wearable electronics and smart textiles. *The J. of The Text. Inst.* **2017**, *108*, 631–652. doi:10.1080/00405000.2016.1177870.
4. Malmivaara, M. The emergence of wearable computing. In *Smart Clothes and Wearable Technology*, McCann, J.; Bryson, B., Eds. Woodhead Publishing Ltd: Cambridge, England, 2009; pp 3–24, doi:10.1533/9781845695668.1.3.
5. International Organization for Standardization. ISO BS EN 9237:1995 Textiles - Determination of the permeability of fabrics to air. In Proceedings of the International Organization for Standardization: Geneva, Switzerland, 1995.
6. Chatterjee, A.; Kumar, M.N.; Maity, S. Influence of graphene oxide concentration and dipping cycles on electrical conductivity of coated cotton textiles. *The J. of The Text. Inst.* **2017**, *108*, 1910–1916. doi:10.1080/00405000.2017.1300209.
7. Ali, A.; Nguen, N.H.A.; Baheti, V.; Ashraf, M.; Militky, J.; Mansoor, T.; Noman, M.T.; Ahmad, S. Electrical conductivity and physiological comfort of silver coated cotton fabrics. *The J. of The Text. Inst.* **2017**, *109*, 620–628. doi:10.1080/00405000.2017.1362148.
8. Varnaite-Zuravliova, S.; Sankauskaite, A.; Stygiene, L.; Krauledas, S.; Bekampiene, P.; Milciene, I. The investigation of barrier and comfort properties of multifunctional coated conductive knitted fabrics. *J. of Ind. Text.* **2016**, *45*, 585–610. doi:10.1177/1528083714564637.
9. International Organization for Standardization. ISO 11092:2014 Textiles - Physiological effects - Measurement of thermal and water-vapour resistance under steady-state conditions (sweating guarded-hotplate test). International Organization for Standardization: Geneva, Switzerland, 2014.
10. International Organization for Standardization. ISO 4920:2012 Textile fabrics - Determination of resistance to wetting (spray test). International Organization for Standardization: Geneva, Switzerland, 2012.
11. American Society for Testing and Materials. ASTM D6767:2016 Standard test method for pore size characteristics of geotextiles by capillary flow test. ASTM International: West Conshohocken, PA, Montgomery, 2016.
12. British Standards Institution. BS 7209:1990 Specification for water vapour permeable apparel fabrics. British Standards Institution: London, UK, 1990.
13. British Standards Institution. BS EN 12127:1998 Textiles - Fabrics - Determination of mass per unit area using small samples. British Standards Institution: London, UK, 1998.
14. British Standards Institution. BS EN 20811:1992 Textiles - Determination of resistance to water penetration - Hydrostatic pressure test. British Standards Institution: London, UK, 1992.
15. International Organization for Standardization. ISO 13934-1:2013 Textiles - Tensile properties of fabrics - Part 1: Determination of maximum force and elongation at maximum force using the strip method. International Organization of Standardization: Geneva, Switzerland, 2013.
16. British Standards Institution. BS EN 14704-1:2005 Determination of the elasticity of fabrics - Part 1: Strip tests. British Standards Institution: London, UK, 2005.
17. British Standards Institution. BS 14704-1:2005 Determination of elasticity of fabrics - Strip tests. British Standards Institution: London, UK, 2005.
18. British Standards Institution. BS 4952:1992 Methods of test for elastic fabrics. British Standards Institution: London, UK, 1992.
19. International Organization for Standardization. ISO 9867:2009 Textiles - Evaluation of the wrinkle recovery of fabric - Appearance method. International Organization for Standardization: Geneva, Switzerland, 2009.
20. International Organization for Standardization. ISO 7854:1995 Rubber or plastics-coated fabrics -- Determination of resistance to damage by flexing. International Organization for Standardization: Geneva, Switzerland, 1995.
21. American Society for Testing and Materials. ASTM D1388-e1:2014 Standard test method for stiffness of fabrics. ASTM International: West Conshohocken, PA, Montgomery, 2014.
22. British Standards Institution. BS 3356:1990 Method for determination of bending length and flexural rigidity of fabrics. British Standards Institution: London, UK, 1990.
23. British Standards Institution. BS 5058:1973 Method for the assessment of drape of fabrics. British Standards Institution: London, UK, 1973.

24. International Organization for Standardization. ISO 12945-2:2000 Textiles - Determination of fabric propensity to surface fuzzing and to pilling - Part 2: Modified Martindale method. International Organization for Standardization: Geneva, Switzerland, 2000.
25. International Organization for Standardization. ISO 12947-2:2016 Textiles - Determination of the abrasion resistance of fabrics by the Martindale method - Part 2: Determination of specimen breakdown. International Organization for Standardization: Geneva, Switzerland, 2016.
26. International Organization for Standardization. ISO 105-X12:2016 Textiles - Tests for colour fastness - Part X12: Colour fastness to rubbing. International Organization for Standardization: Geneva, Switzerland, 2016.
27. International Organization for Standardization. ISO 3759:2011 Textiles - Preparation, marking and measuring of fabric specimens and garments for determination of dimensional change. International Organization for Standardization: Geneva, Switzerland, 2011.
28. International Organization for Standardization. ISO 6330:2012 Textiles - Domestic washing and drying procedures for textile testing. International Organization for Standardization: Geneva, Switzerland, 2012.
29. Matsouka, D.; Vassiliadis, S.; Tao, X.; Koncar, V.; Bahadir, S.K.; Kalaoglu, F.; Jevsnik, S. Electrical connection issues on wearable electronics, IOP Conference Series: Materials Science and Engineering, Levos, Greece, 5–7 September, 2018, 2018; IOP Publishing, doi:10.1088/1757-899X/459/1/012017.
30. International Organization for Standardization. ISO 105-C10:2006 Textiles - Tests for colourfastness - Part C10: Colour fastness to washing with soap or soap and soda. International Organization for Standardization: Geneva, Switzerland, 2006.
31. International Organization for Standardization. ISO 105-C06:2010 Textiles - Tests for colour fastness - Part C06: Colour fastness to domestic and commercial laundering. International Organization for Standardization: Geneva, Switzerland, 2010.
32. Varesano, A.; Tonin, C. Improving electrical performances of wool textiles: synthesis of conducting polypyrrole on the fiber surface. *Text. Res. J.* **2008**, *78*, 1110–1115. doi:10.1177/0040517507077488.
33. Karim, N.; Afroj, S.; Tan, S.; He, P.; Fernado, A.; Carr, C.; Novoselov, K.S. Scalable production of graphene-based wearable e-textiles. *Am. Chem. Soc. Nano* **2017**, *11*, 12266–12275. doi:10.1021/acsnano.7b05921.
34. American Association of Textile Chemists and Colorists. AATCC 132:2013 Colorfastness to drycleaning. American Association of Textile Chemists and Colorists: Research Triangle Park, North Carolina, USA, 2013.
35. Wu, B.; Zhang, B.; Wu, J.; Wang, Z.; Ma, H.; Yu, M.; Li, L.; Li, J. Electrical switchability and dry-wash durability of conductive textiles. *Sci. Rep.* **2015**, *5*. doi:10.1038/srep11255.
36. American Association of Textile Chemists and Colorists. AATCC 86:2016 Drycleaning: Durability of applied designs and finishes. American Association of Textile Chemists and Colorists: Research Triangle Park, North Carolina, USA, 2016.
37. American Association of Textile Chemists and Colorists. AATCC Test method 61-2013 Colorfastness to Laundering: Accelerated. American Association of Textile Chemists and Colorists: Research Triangle Park, North Carolina, USA, 2013.
38. Hu, X.; Tian, M.; Qu, L.; Zhu, S.; Han, G. Multifunctional cotton fabrics with graphene/polyurethane coatings with far-infrared emission, electrical conductivity, and ultraviolet-blocking properties. *Carbon* **2015**, *95*, 625–633. doi:10.1016/j.carbon.2015.08.099.
